# Infectious sacroiliitis due to group A streptococcus infection during pregnancy: a case report

**DOI:** 10.1186/s13256-022-03271-4

**Published:** 2022-02-11

**Authors:** Mikako Nagashima, Norikazu Watanabe, Yosuke Okui, Mika Fukase, Kanako Takahashi, Tsuyoshi Ohta, Seiji Tsutsumi, Yuya Takakubo, Michiaki Takagi, Satoru Nagase

**Affiliations:** 1 Department of Obstetrics and Gynecology, Yamagata University Faculty of Medicine, 2-2-2 Iida-Nishi, Yamagata, 990-9585 Japan; 2 Department of Rehabilitation, Yamagata University Faculty of Medicine, Yamagata, Japan; 3 Department of Orthopedic, Yamagata University Faculty of Medicine, Yamagata, Japan

**Keywords:** Group A streptococcus, Pregnancy, Sacroiliitis, Toxic shock syndrome, Case report

## Abstract

**Background:**

Group A streptococcus infection during pregnancy can be concerning. It may cause toxic shock syndrome, which can be fatal. Here, we report a rare case of a pregnant woman who developed infectious sacroiliitis due to group A streptococcus infection. To the best of our knowledge, this case is the first of its kind to be reported.

**Case presentation:**

A 32-year-old multiparous Japanese woman presented with fever and right buttock pain at 28 weeks of gestation. Based on our clinical findings and investigations, she was diagnosed with group A streptococcus bacteremia and infectious sacroiliitis caused by group A streptococcus. A cardiotocography performed to assess the fetal status showed fetal tachycardia. To prevent the patient from progressing to toxic shock syndrome caused by group A streptococcus, we performed an emergency cesarean section. The patient and her infant had a good course after the cesarean section.

**Conclusion:**

A pregnant woman diagnosed with group A streptococcus infection needs to be monitored closely because a timely decision to deliver the fetus before rapid deterioration to toxic shock syndrome is crucial.

## Background

Group A streptococcus (GAS) bacteria are commensal human organisms. However, they may cause toxic shock syndrome (TSS), an extremely fatal disease leading to circulatory failure, respiratory failure, and disseminated intravascular coagulation that could result in multiple organ failure within 24 hours of its onset. The clinical course of TSS due to GAS infection in pregnancy tends to be severe. According to a report summarizing maternal deaths in Japan from 2010 to 2013, approximately 4% of maternal deaths were caused by GAS-TSS (TSS caused by group A streptococcus). Two-thirds of pregnant women who developed fulminant infection died within one day [1]. Moreover, myositis of the uterus induced by GAS causes strong uterine contractions resulting in intrauterine fetal death and stillbirth [2]. Therefore, GAS infection in pregnant women is a matter of grave concern. Regardless of past experiences with perinatal GAS infection, the diagnostic criteria and treatment methods have not been sufficiently established yet. Infectious sacroiliitis in pregnancy is rare and difficult to differentiate from noninfectious back pain associated with pregnancy because of the nonspecific nature of the symptoms. Here, we report a rare case of infectious sacroiliitis during pregnancy caused by GAS. To the best of our knowledge, this is the first report of such a case.

## Case presentation

A 32-year-old Japanese woman, gravida two and para one, at 28 weeks of gestation, developed a fever of over 38 °C following right buttock pain without any known cause after waking up. She was brought to our emergency room for increasing pain. A brief history revealed she was being treated with certolizumab pegol for rheumatoid arthritis for 2 years. Her rheumatoid arthritis was graded as Stage II by Steinbrocker’s classification, and she had no lesions on her sacroiliac joints (Larsen grade 0). She was also suspected of suffering from Sjogren’s syndrome and experienced upper respiratory symptoms 2 weeks before the onset of buttock pain.

On initial clinical assessment, her temperature was 37.8 °C and her heart rate was 107 beats per minute. She had no respiratory or abdominal symptoms. She complained of a sharp pain around her right sacroiliac joint that was tender on examination.

**Fig. 1:**
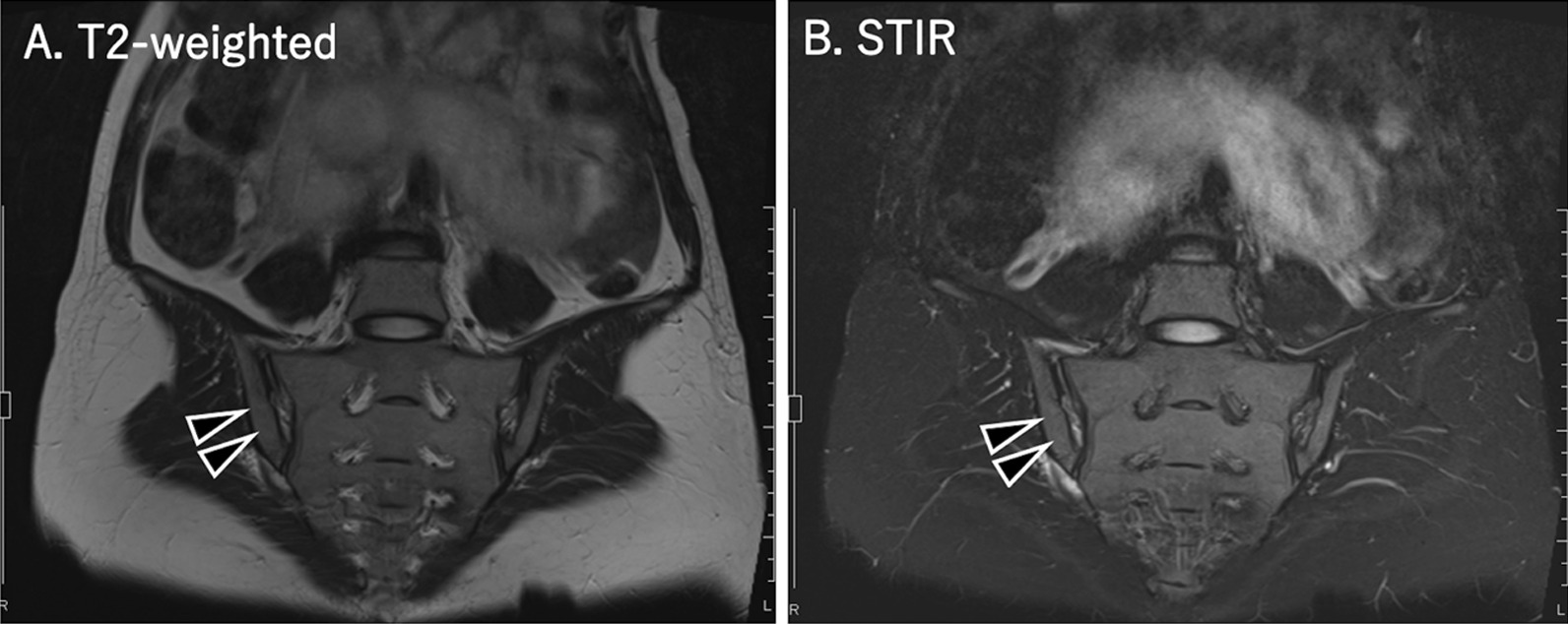
Magnetic resonance imaging scans of the sacroiliac joints of the pregnant patient with right-sided infectious sacroiliitis at the time of hospitalization. **A** T2-weighted (T2W), **B** Short-tau inversion recovery (STIR). Arrow head shows high-intensity signal area in the right sacroiliac joint

Laboratory investigations revealed that her white blood cell count (17,800/μL, normal value < 7800/μL) and C-reactive protein (2.63 mg/dL, normal value < 0.3 mg/dL) values were high at the time of hospitalization. An obstetric sonography showed that she did not have a thickened placenta or any retroplacental collection. The estimated fetal weight was 1245 g. Her right sacroiliac joint demonstrated a high-intensity signal on both T2-weighted magnetic resonance imaging (MRI) and short-tau inversion recovery (STIR), which suggested the presence of arthritis (Fig. 1). She was diagnosed with right infectious sacroiliitis and admitted to our orthopedic department. Her treatment was initiated with intravenous administration of ceftriaxone (2 g per day). The next day, she complained of decreased fetal movement. A cardiotocography showed fetal tachycardia (180–190 beats per minute). Furthermore, GAS was detected in her blood culture collected on admission. Urine, nasal, and pharyngeal cultures were all negative for GAS. We diagnosed her with GAS bacteremia and performed an emergency cesarean section under general anesthesia. She delivered a female neonate weighing 1286 g with Apgar scores of 3 and 7 at 1 and 5 minutes, respectively. Her umbilical artery pH was 7.344.

**Fig. 2:**
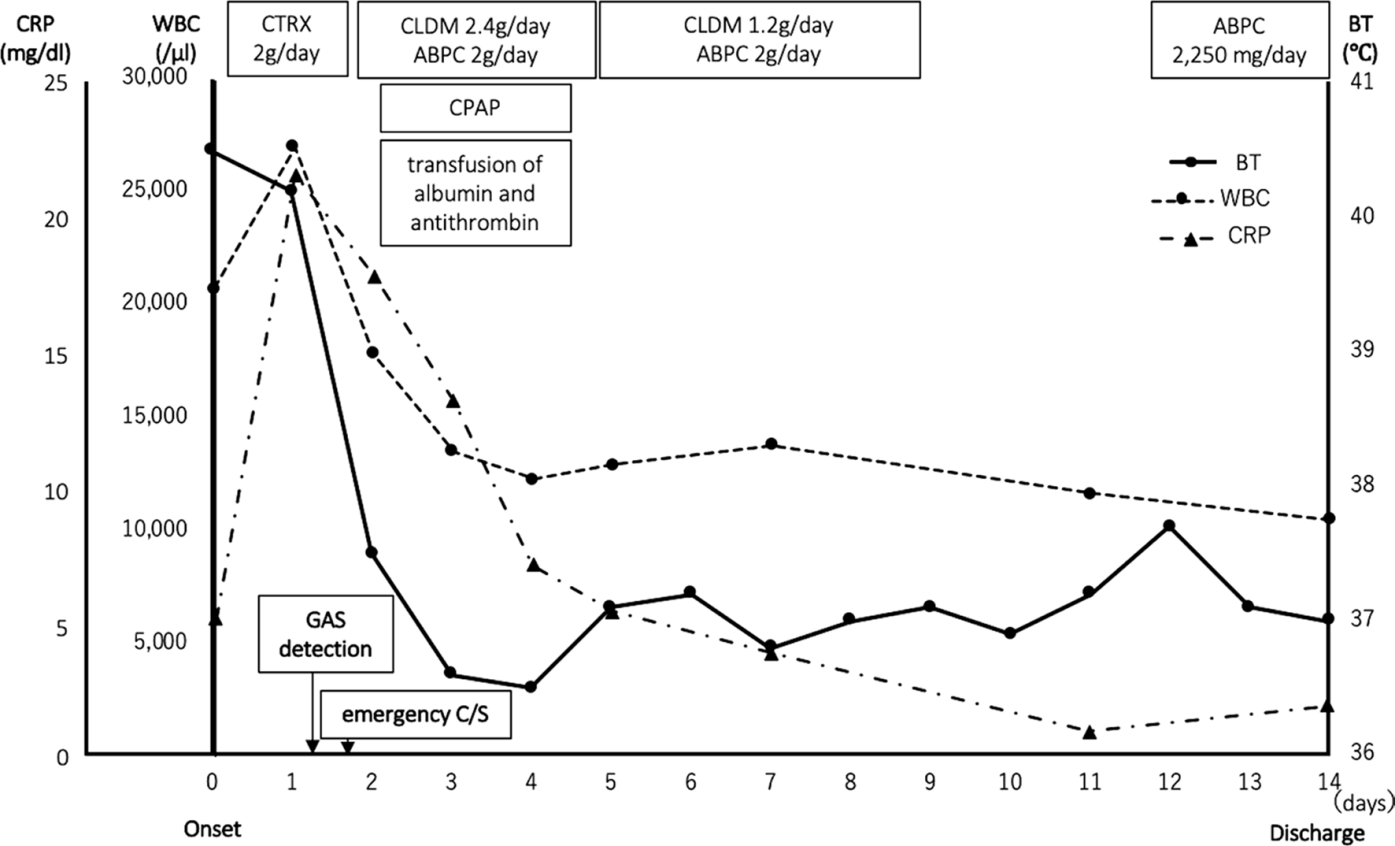
Clinical course of the pregnant patient with infectious sacroiliitis caused by group A streptococcus. ABPC, ampicillin; BT, body temperature; CLDM, clindamycin; CPAP, continuous positive airway pressure; CRP, C-reactive protein; C/S, cesarean section; CTRX, ceftriaxone; GAS: group A streptococcus, WBC: white blood cell count

After the operation, the patient was moved to an intensive care unit. Ceftriaxone administration was stopped after a day, and her antibiotics were changed to clindamycin (2.4 g per day) and ampicillin (2 g per day) to treat the GAS infection. Her blood gas analysis showed poor oxygenation, and chest x-ray showed mild pulmonary edema; therefore, she was continued on oxygen administration with a venturi mask. Next day post-surgery, her blood tests revealed low albumin (1.7 g/dL) and antithrombin (61%) levels. Hence, an albumin preparation and antithrombin was administered. On the same day, her chest x-ray showed exacerbation of the pulmonary edema. She was then started on continuous positive airway pressure for 2 days. Three days after surgery, she was moved to the general ward once she was stable. Over the following days, the patient progressively improved, and her intravenous antibiotics were discontinued. She was started on an oral administration of amoxicillin (2250 mg per day) and discharged after a 15-day hospital stay (Fig. 2). Her sacroiliac joint was rated Larsen grade 0 (normal) upon x-ray after discharge.

A pathological examination of her placenta revealed no evidence of chorioamnionitis, corditis, placental infarction, or placental hemangiomatosis. However, villitis of unknown etiology was partially recognized.

The female baby was evaluated by a neonatologist, and diagnosed with respiratory distress syndrome. She was intubated and administered a surfactant. She had no obvious signs of infection and was treated for extremely low birth weight in the neonatal intensive care unit. Administration of ampicillin (100 mg/kg/day) was initiated as a preventive measure for 4 days. Her blood culture was negative. She was extubated 4 days after birth and discharged from the hospital 81 days after birth. Her weight was 3172 g at discharge.

## Discussion and conclusions

Pregnancy-related infectious sacroiliitis has a very low incidence. It has been reported to occur during pregnancy, in the postpartum period, or after a miscarriage. During pregnancy, the sacroiliac joints relax and receive increased blood flow, increasing the risk of hematogenous infection. Pressure from an enlarged uterus or damage caused to sacroiliac joints during delivery can further increase this risk. These changes in pregnant women are thought to initiate the pathogenic mechanism of pregnancy-related sacroiliitis [3]. Although the causative bacterium is not always detected, *Staphylococcus aureus* is the most common cause of pregnancy-related infectious sacroiliitis [4]. To the best of our knowledge, this is the first reported case of sacroiliitis due to GAS infection during pregnancy. Besides this case, only one other case of sacroiliitis caused by GAS in the postpartum period has been reported [5]. Peripartum infectious sacroiliitis due to GAS is rare and difficult to diagnose.

It is important to consider noninfectious sacroiliitis as a differential diagnosis in such cases because diagnosis of an infection localized at the sacroiliac joint is difficult. Our patient had been known to suffer from rheumatoid arthritis. Sacroiliitis associated with rheumatoid arthritis is found to occur in about 30% of patients with Steinbrocker’s stage III–IV; however, it is less common in patients with stage I–II [6]. Our patient was stage II. Since she had no lesions on her sacroiliac joints and her onset of symptoms was rapid, sacroiliitis due to rheumatoid arthritis was excluded. Considering her pregnancy, we avoided a joint aspiration and arrived at a diagnosis based on her symptoms and her MRI report. We finally confirmed our diagnosis as sacroiliitis due to GAS infection based on the blood culture report.

A number of criteria for diagnosing GAS-TSS have been proposed by the Centers for Disease Control and Prevention [7]. In Japan, a GAS-TSS diagnostic criteria draft from the Ministry of Health and Welfare research group has been presented [8]. Regardless of these reports, the diagnostic criteria for GAS-TSS have not yet been established in Japan. Perinatal GAS-TSS, especially in late pregnancy, is characterized by rapid progression [2]. GAS-TSS in pregnancy is rapidly exacerbated because the bacteria growing in the myometrium are pushed into the bloodstream by frequent contractions of the uterus, which is not the case in GAS-TSS occurring in nonpregnant and postpartum women.

An early diagnosis of GAS infection is difficult because the initial symptoms of GAS-TSS, such as upper respiratory inflammation, are atypical. Our patient also had upper respiratory symptoms 2 weeks before her admission. A rapid antigen detection test would have been useful for early diagnosis of GAS infection in our patient; however, it was not performed as we did not suspect GAS infection at admission. We observed that, in the clinical course of such patients, an abnormal fetal heart rate tends to precede maternal and fetal deterioration.

The protocol for delivery in pregnant patients with perinatal GAS infection remains unclear. In general, strategies such as the administration of antibiotics and systemic treatment against shock and multiple organ failure are followed. However, the optimal timing and method of delivery have not been determined. This case was diagnosed as a GAS infection but did not progress to TSS. We performed an emergency cesarean section when we detected GAS in the blood culture and observed fetal tachycardia to prevent an exacerbation to GAS-TSS of the “parturition type.” If infectious sacroiliitis is caused by bacteria other than GAS, the estimated prognosis is not poor. However, our patient was determined to have GAS infection manifesting as sacroiliitis and bacteremia, which could rapidly exacerbate GAS-TSS and cause poor prognosis. During pregnancy, we consider treatment before the development of myometrial GAS infection as important, because the patients could have poor prognoses once GAS-TSS parturition type develops. Since no uterine contractions were observed preoperatively and no bacterial colonization was detected in the placenta post-delivery, we concluded that there was no GAS proliferation in the myometrium. Based on the fact that she did not have premature uterine contractions, we thought that early delivery by cesarean section could prevent her from progressing to the parturition type of GAS-TSS.

Here, we presented a rare case of a pregnant woman with infectious sacroiliitis caused by GAS at 28 weeks of gestation. As there is no guideline to manage women with GAS infection during pregnancy, based on our patient’s clinical course, we decided to perform a cesarean section to prevent her progression to a serious condition regardless of her pregnancy  being at a preterm gestational stage.

Thus, we conclude that, when a pregnant woman develops GAS bacteremia, early delivery can be considered to prevent a rapid progression to the “parturition type” of GAS-TSS that could be fatal for the mother and her infant.

## Data Availability

Not applicable.

## Abbreviations

GAS: Group A streptococcus
TSS: Toxic shock syndrome
GAS-TSS: Toxic shock syndrome caused by group A streptococcus
MRI: Magnetic resonance imaging
STIR: Short-tau inversion recovery
